# Data-independent Acquisition Improves Quantitative Cross-linking Mass Spectrometry*[Fn FN2]

**DOI:** 10.1074/mcp.TIR118.001276

**Published:** 2019-01-16

**Authors:** Fränze Müller, Lars Kolbowski, Oliver M. Bernhardt, Lukas Reiter, Juri Rappsilber

**Affiliations:** From the ‡Bioanalytics, Institute of Biotechnology, Technische Universität Berlin, 13355 Berlin, Germany;; §Wellcome Centre for Cell Biology, School of Biological Sciences, University of Edinburgh, Edinburgh EH9 3BF, Scotland, United Kingdom;; ¶Biognosys, 8952 Zürich-Schlieren, Switzerland

**Keywords:** Protein Cross-linking*, Label-free quantification, Mass Spectrometry, Quantification, Bioinformatics software, Data independent acquisition, Reproducibility, Spectronaut, Xi

## Abstract

Quantitative cross-linking mass spectrometry (QCLMS) reveals structural detail on altered protein states in solution. On its way to becoming a routine technology, QCLMS could benefit from data-independent acquisition (DIA), which generally enables greater reproducibility than data-dependent acquisition (DDA) and increased throughput over targeted methods. Therefore, here we introduce DIA to QCLMS by extending a widely used DIA software, Spectronaut, to also accommodate cross-link data. A mixture of seven proteins cross-linked with bis[sulfosuccinimidyl] suberate (BS^3^) was used to evaluate this workflow. Out of the 414 identified unique residue pairs, 292 (70%) were quantifiable across triplicates with a coefficient of variation (CV) of 10%, with manual correction of peak selection and boundaries for PSMs in the lower quartile of individual CV values. This compares favorably to DDA where we quantified cross-links across triplicates with a CV of 66%, for a single protein. We found DIA-QCLMS to be capable of detecting changing abundances of cross-linked peptides in complex mixtures, despite the ratio compression encountered when increasing sample complexity through the addition of *E. coli* cell lysate as matrix. In conclusion, the DIA software Spectronaut can now be used in cross-linking and DIA is indeed able to improve QCLMS.

## Abstract (precis)

We present here a simple, user friendly and automated new quantitative cross-linking mass spectrometry (QCLMS) workflow comprising data-independent acquisition (DIA) for acquiring mass spectrometry data and Spectronaut, one of the leading DIA analysis tools. DIA cross-linking data outperforms DDA in reproducibility and accuracy of quantitation results. DIA-QCLMS tolerates complex backgrounds and through its automation recommends itself for routine application in the analysis of protein complex dynamics.

## Graphical Abstract

**Figure:**
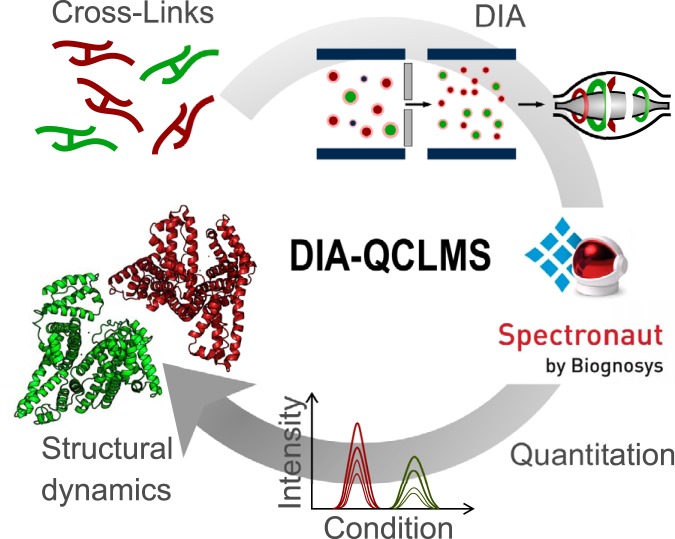

**Highlights**

- Quantitative cross-linking mass spectrometry (QCLMS) was automated by Spectronaut.
- Data-independent acquisition (DIA) was adapted to QCLMS.
- Accuracy and precision of quantitation improves with DIA over DDA.
- QCLMS is now ready for use in complex samples.

Cross-linking mass spectrometry (CLMS)^1^ is a powerful tool for studying the 3D structure of proteins and their complexes (1–5). Chemical cross-linking helps to identify residue pairs that are in proximity in native structures but not necessarily in primary sequence, by introducing covalent bonds between these residues. Subsequent to the cross-linking reaction and the proteolytic digestion of proteins, cross-linked peptides can be enriched (using strong cation exchange (SCX) (6) or size exclusion chromatography (SEC) (7), for example) and then identified through liquid chromatography-mass spectrometry (LC-MS) combined with database searching.

Although a protein's function links to its three-dimensional structure, these structures are intrinsically dynamic and can change (8, 9). Adding quantitative information to the relative abundances of cross-linked residue pairs offers a unique opportunity to study the structural flexibility and changes of proteins (10). Previous studies using quantitative cross-linking mass spectrometry (QCLMS) have provided concepts and techniques for studying changing protein states including activation (11), regulation of protein networks (12–15), maturation of complexes (16), regulation of enzyme activity (17–19), protein-protein interactions (20, 21) and interactome analysis of cancer cell lines (22).

Broadly speaking, two quantitative strategies are suitable for QCLMS: labeled and label-free. Although isotope-labeled cross-linkers (23) are commonly used in labeling strategies (13, 14, 16–19, 24–29), other general strategies have also been adapted to QCLMS including SILAC (stable isotope-labeled amino acids) (22, 30, 31) and isobaric labeling by TMT (32, 33) or iTRAQ (34). In contrast, label-free quantitation (LFQ) might allow for a simpler experimental design and reduced costs. Importantly, although samples are processed separately during LFQ experiments, which may increase technical variance, label-free QCLMS is as reproducible as other proteomic techniques (35).

Multiple approaches are used in proteomics for LFQ (36, 37). Data-dependent acquisition (DDA) unfortunately results in poor reproducibility for low abundance proteins or peptides (38–40) and therefore is not ideal for the typically low abundance cross-linked peptides. Targeted proteomic strategies such as SRM (MRM) or PRM excel for less abundant peptides (41–45). Early targeted approaches on cross-linking mass spectrometry using an inclusion list were performed by Barysz *et al.* 2015 (46) and more recently, on MS2 level using parallel reaction monitoring (PRM) and Skyline (47). However, the number of targets is limited, and the analysis is demanding. Data-independent acquisition (DIA) promises a solution to all these challenges by requiring minimal assay development and allowing large scale quantitative analysis with high reproducibility (48, 49). This has not yet been exploited in QCLMS because of current software restrictions regarding cross-linked peptides.

In recent years, significant advances in software for both CLMS and QCLMS have propelled the cross-linking field forward, enabling a deeper understanding of dynamic protein systems and a wider range of workflows (50). Here, we developed a DIA-QCLMS workflow that uses the Spectronaut software for the quantitation of observed unique residue pairs. We determined the accuracy and reproducibility of our DIA-QCLMS workflow at both MS1 as well as MS2 level, using a mix of seven proteins, each cross-linked using bis[sulfosuccinimidyl] suberate (BS^3^), and *E. coli* cell lysate as matrix.

## EXPERIMENTAL PROCEDURES

### 

#### 

##### Reagents

The seven-protein mix comprised human serum albumin (HSA), cytochrome C (bovine heart), ovotransferrin (Conalbumin, chicken egg white), myoglobin (equine heart), lysozyme C (chicken egg white), and catalase (bovine liver), all purchased individually from Sigma Aldrich (St. Louis, MO). Creatine kinase Type M (rabbit muscle) was purchased from Roche (Basel, Switzerland). The cross-linker BS^3^ was purchased from Thermo Scientific Pierce (Rockford, IL).

##### Cross-linking Reaction

Cross-linking reactions of the individual proteins were performed in parallel as previously described (35). In short, purified proteins were mixed separately with BS^3^ (1 μg/μl protein concentration), with a protein to cross-linker mass ratio of 1:4. After incubation on ice, the reaction was stopped using saturated ammonium bicarbonate. Cross-linked proteins were subjected to SDS-PAGE, visualized using Coomassie staining and monomer bands were excised for digestion.

##### Sample Preparation

Cross-link protein gel bands were reduced, alkylated and digested using trypsin as described before (51). After digestion, peptides were extracted from gel bands using 80% v/v acetonitrile (ACN) and concentrated to a final ACN content of nominally 5% v/v using a Vacufuge Concentrator (Eppendorf, Germany). Tryptic peptides were enriched using strong cation exchange chromatography (SCX) as previously described (6) but using SCX-StageTips (52, 53) with minor modifications for activation of the Tip and gradient steps. The SCX-StageTips were activated using first methanol, following buffer 2 (0.5% AcH, 80% CAN), buffer 1 (0.5% AcH), high salt buffer (0.5% AcH, 20% CAN, 600 mm NH_4_Ac) and finally again buffer 1. Peptides were eluted in steps using: 50 mm NH_4_Ac (fraction 1), 100 mm NH_4_Ac (fraction 2), 200 mm MH_4_Ac (fraction 3), 300 mm NH_4_Ac (fraction 4), 500 mm NH_4_Ac (fraction 5), 600 mm NH_4_Ac (fraction 6). Peptides were then desalted using C_18_-StageTips (52, 54) and eluted using 80% v/v ACN, 0.1% v/v TFA. Peptides were dried down and resuspended in 2% v/v ACN, 0.1% v/v formic acid (FA) to a final protein concentration of 0.75 μg/μl.

##### Data Acquisition

LC-MS/MS analysis was performed using a tribrid Orbitrap mass spectrometer (Orbitrap Fusion™ Lumos, Thermo Fisher Scientific, CA) with a “high/high” (high-resolution MS1 and MS2) acquisition strategy. 1.5 μg peptides were injected for data-dependent acquisition (DDA) experiments. For data-independent acquisition (DIA), the stock solution (1.5 μg peptides) was diluted to reach 0.1×, 0.3×, 0.5×, 0.7×, 0.9×, and 1× (undiluted). 1.5 μg tryptic *E. coli* cell lysate was added as matrix to each sample of the dilution series to assess DIA in the context of analyzing a complex sample. iRT peptides (Biognosys, Switzerland) were added to each sample before MS acquisition. The peptide separation was carried out on an EASY-Spray column (50 cm × 75 μm ID, PepMap C_18_, 2 μm particles, 100 Å pore size, Thermo Fisher Scientific, Germany). Peptides were separated using a 150 min gradient and analyzed in DDA mode as described before (35). In short, precursor ions were detected in the Orbitrap at 120K resolution using *m/z* range 400–1600. Ions with charge states from 3+ to 7+ were selected for fragmentation. Selected ions were isolated and fragmented by high energy collision dissociation (HCD) and detected in Orbitrap at 30K resolution (55). In DIA mode, precursor ions were acquired using a MS1 master scan (*m/z* range: 400–1200, max. injection time: 60 ms, AGC target: 4 × 10^5^, detector: Orbitrap, resolution: 60K), following 66 DIA scans for MS2 within a fragmentation rage of *m/z* 120–1200 using an isolation window width of *m/z* 12 and a max. injection time of 50 ms. Selected ions were isolated in the quadrupole, fragmented using HCD (normalized collision energy 30%) and detected in Orbitrap at 30K resolution.

##### Identification of Cross-linked Peptides

The raw mass spectrometric data files were processed into peak lists using MaxQuant (56) (v. 1.5.0.0) as described previously (35). Xi (v. 1.6.723) (57) was used for database search. The database comprised the sequences of HSA (UniProt ID: P02768), cytochrome C (P62894), ovotransferrin (P02789), myoglobin (P68082), creatine kinase (P00563), lysozyme C (P00698), and catalase (P00432) and the reverse sequence of each of these proteins as decoys. Search parameters were: MS tolerance: 6 ppm, MS/MS tolerance: 20 ppm, enzyme: trypsin, missed cleavages: 4, cross-linker: BS^3^, fixed modification: carbamidomethylation of cysteine, variable modification: oxidation of methionine and modification by BS^3^ with the second NHS ester hydrolyzed or amidated, with BS^3^ reaction specificity at lysine, serine, threonine, tyrosine and N termini of proteins. In a cross-link analysis, the false discovery rate (FDR) can be calculated on different information levels: PSMs, peptide pairs, residue pairs (RPs) and protein pairs (58). We here considered residue-pair FDR, which were estimated using xiFDR (v 1.0.21.45) with the equation: FDR = TD-DD/TT (58) and filtering to only use cross-link PSMs within proteins. The max. protein ambiguity was set to 1. Other settings were left on default. Identification with 5% FDR at link level were accepted for quantitation.

##### Creation of Spectral Library for Cross-links and Quantitation

Quantitation was performed on MS1 and MS2 level using Spectronaut (version 11.0.15038.23.25164) (59, 60). The spectral library of cross-linked peptides was introduced as a .csv file, following the standard format for custom libraries in Spectronaut (Manual for Spectronaut 11, available on Biognosis website). The .csv file was constructed from our DDA data using xiDIA-library (a Python script generated by us for this purpose). xiDIA-library is an open source collaborative initiative available in the GitHub repository https://github.com/Rappsilber-Laboratory/xiDIA-library. It is freely available under the Apache License v2.0. xiDIA-library extracts the required information for the spectrum library from different sources: Precursor information (for example, *m/z*, charge) are read from the xiFDR PSM result file; fragment data is obtained through annotation by xiAnnotator [https://github.com/Rappsilber-Laboratory/xiAnnotator]; retention times are extracted from mzML files, obtained by subjecting raw files to MSconvert (61); iRT values are determined using linear regression of observed retention times of iRT peptides. Up to 10 cross-link containing fragments and up to 10 linear ones were chosen from the highest intensity b- or y-ion signals in the *m*/*z* range 300–1400. The library was imported as an external library, leaving out the Prepare Perspective option in Spectronaut (called Library in Spectronaut 12). Note, for optimal import use the “set up a DIA Analysis from File” option in the View tab (Analysis tab in Spectronaut 12) and follow the wizard. Spectronaut 11 and Spectronaut 12 show the same performance in analyzing cross-linking data (see supplemental Fig. S1).

Protein modifications must be defined in Spectronaut to enable internal decoy generation for quantitation. The following cross-linker modifications were added manually to the default list of modifications in Spectronaut: BS^3^-OH (156.078 Da), BS^3^-NH_2_ (155.094 Da), and BS^3^-d0 (138.068 Da). Defaults were used for the remaining settings. MS1 and MS2 filtering was done according to the Spectronaut manual with the following deviations: Quantitation tab: Interference correction unticked, Minor (Peptide) Grouping: by modified sequence, Major Group Top N unticked, Minor Group Top N ticket (max 10, min 1), Minor Group Quantity: mean precursor quantity. Note that the interference correction in Spectronaut works only for complex mixtures with a background proteome library (linear peptides). Interference correction requires matching sequences from the spectral library to the FASTA file supplied for the analysis. This is currently not possible with cross-linked peptides. After automated peak-picking and retention time alignment of Spectronaut, a manual correction of peak boundaries was performed for cross-linked peptides with a coefficient of variation (CV) above 30% using the filter option “condition CV” in the View Perspective. Data from Spectronaut was exported using the Report Perspective into a .xls file to integrate feature-level quantitation data into residue-level data using a standard spreadsheet application (Excel, Microsoft).

The background *E. coli* Library (linear peptides) was generated using MaxQuant for linear search with default settings and the Prepare Perspective option in Spectronaut (default setting).

Unfortunately it is not possible to use Spectronaut to perform DDA quantitation analysis, hence we had to use Skyline (62) (v. 4.2) to compare DIA and DDA quantitation results. Creation of Spectral library and quantitation settings in Skyline are as described previously (35). Shortly, the .ssl file for custom libraries in Skyline was constructed using an in-house-script based on peptide spectrum matches (PSM) of identified cross-linked peptides. The library file and the assigned mzML files are used to create the final spectral library within Skyline. The following modifications had to be defined in Skyline: cross-linker (25.968 Da), BS^3^-OH (156.078 Da), BS^3^-NH_2_ (155.094 Da) and BS^3^-loop (138.068 Da), linkage site (1.0078 Da). The Spectral library is used to match precursor information of identified precursors to the DDA data. Note that quantitation in Skyline can currently only be performed on MS1 level for many targets. After quantitation, the data were exported into a .csv file and feature-level data were integrated into residue-level using Excel. CV values within replicates (triplicates) had to be calculated separately for each unique residue pair using the following equation: c_v_ = σ/μ.

##### Experimental Design and Statistical Rationale

For DIA MS experiments, we analyzed triplicates of a pooled seven-protein mixture in a dilution series of 0.1×, 0.3×, 0.5×, 0.7×, 0.9×, and 1× stock solution. Each dilution was injected three times, resulting in 18 LC-MS injections (DDA: 15 injections, missing 0.7×). A second dilution series of the same steps as before was mixed with tryptic *E. coli* cell lysate as matrix and injected as described above. Hence, 36 individual DIA runs were analyzed in total for this study. Peak areas were quantified in Spectronaut and Skyline. Conducting a dilution series prevented the mean peak intensity in the samples to be used for data normalization. Thus, nonnormalized data was used. Only cross-linked peptides quantified in three out of three technical replicas (triplicates) were considered for label-free quantitation. Statistical testing was performed using Spectronaut with default settings for statistical tests. A q value of 0.01 was used for filtering the results. The peak selection and boundaries of cross-linked peptides above a CV value of 30% was corrected manually (this applied to approximately one in four PSMs). It should be noted that the peak selection and boundaries in the DDA data were not corrected manually.

## RESULTS AND DISCUSSION

### 

#### 

##### Construction of Spectral Library

**Fig. 1.**
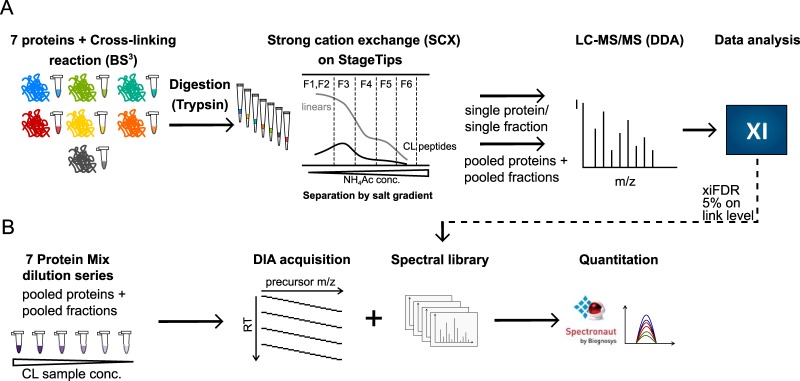
**Label-free DIA-based cross-linking quantitation workflow.** *A*, Sample preparation workflow using SCX chromatography to fractionate cross-linked peptides. *B*, Cross-link identification and quantitation workflow using Xi Software for identification and Spectronaut for quantitation.

First, using data-dependent acquisition (DDA), we generated a library of fragmentation spectra for data-independent acquisition (DIA) analysis. Our sample comprised seven proteins (HSA, cytochrome C, ovotransferrin, myoglobin, creatine kinase, lysozyme, and catalase), each cross-linked separately in solution using BS^3^. We prevented cross-links between proteins from entering our analysis and retained the option to evaluate our identified cross-links against available 3D structures of the seven proteins by only exercising protein monomer SDS-PAGE bands for trypsin digestion. We fractionated and enriched the cross-link peptides of each protein into six SCX-StageTip fractions (6, 52). Each fraction was analyzed individually (totaling 49 runs) and pooled across all proteins and fractions (12 runs) by LC-MS using a “high-high” (high-resolution MS1 and MS2) strategy and DDA (Fig. 1*A*). The analysis yielded 414 unique residue pairs (URPs) across all seven proteins at 5% link level FDR, compared with 83 URPs as seen previously (63). Of the 414 URPs, all were covered by crystallographic protein models, with 350 falling below 30 Å, and 64 (15%) above. The long-distance links did not distribute equally among the seven proteins. For example, in HSA (66 kDa, 137 links), we observed 6% long-distance links whereas we encountered 23% in the similar-sized ovotransferin (76 kDa, 177 links). This indicates conformational flexibility rather than false identifications as the cause of the relatively high fraction of long-distance links. To further increase the library size, we included a public data set from our laboratory (PXD008550), which was generated using size exclusion chromatography with the same seven proteins, cross-linker, and protease. This added 121 URPs to the library including 16 long-distance links and thus did not change the overall match to the structures. We consider cross-linking at K, S, T, Y and protein N termini. 270 URPs included at least one S, T, or Y whereas 265 URPs included only K or N termini (1 URP). The spectral library was then generated using xiDIA-library (see Experimental Procedures) and comprised 535 URPs, 2344 precursors and 34531 fragments in total. Our raw data, peak files and results files are accessible in the ProteomeXchange (64) Consortium via the PRIDE (65) partner repository with the data set identifier PXD011036.

##### Label-free Quantitation of Cross-linked Peptides by Spectronaut Using Data-Independent Acquisition

**Fig. 2.**
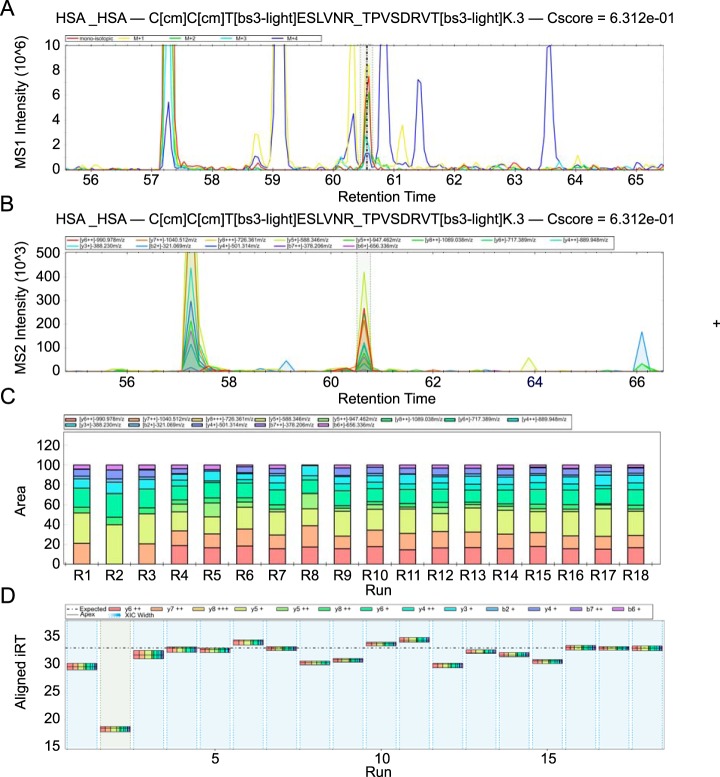
**Visualization of cross-linking features in Spectronaut software.** *A*, MS1 isotope envelope XIC plot, showing extracted precursor ions M (red), M+1 (yellow), M+2 (green), M+3 (light blue), M+4 (dark blue). *B*, MS2 XIC plot, showing extracted fragment ion chromatograms in different colors. *C*, MS2 intensity alignment plot, showing normalized fragment intensities across all replica. *D*, Cross run RT accuracy plot, showing retention time comparison between all replica.

To assess the reproducibility of data-independent acquisition quantitation, we used the pooled sample of proteins and fractions for a dilution series experiment at 0.1×, 0.3×, 0.5×, 0.7×, 0.9×, and 1× of the stock mixture (Fig. 1*B*). iRT peptides were added to all samples to be used as the internal standard for retention time alignment in Spectronaut. Our DIA method (see Experimental Procedures) resulted in a cycle time of 5 s, leading to 14 data points per MS1 peak and 4 data points in MS2, on average. The low number of MS2 data points is caused by the small window size (*m*/*z* 12), that may limit the accuracy of our analysis in MS2. However, the small window size also reduces interferences by co-eluting precursors and hence increases sensitivity for low abundant cross-linked peptides. Analyzing cross-link DIA data required several changes to Spectronaut. The parsing rules were expanded to use cross-link specific information noted in the peptide comments column and the output file was expanded by an URP column (called “FG_Comment”). The work also required several software adjustments to be made (see release notes for Spectronaut version 11.0.15038.23.25164). Spectronaut then successfully read in the cross-link DIA data, conducted retention time alignment and extracted precursor and fragment information based on the external spectral library (Fig. 2). Data was filtered to a q value of 0.01 (comparable to 1% FDR) (66–68). We manually inspected and corrected peak boundaries for all precursor and fragment species with a coefficient of variation (CV) above 30% within replicates (this was the case for about one in four PSMs, taking about 2–3 min per PSM). The quantitation results were then exported using the Report Perspective option in Spectronaut.

**Fig. 3.**
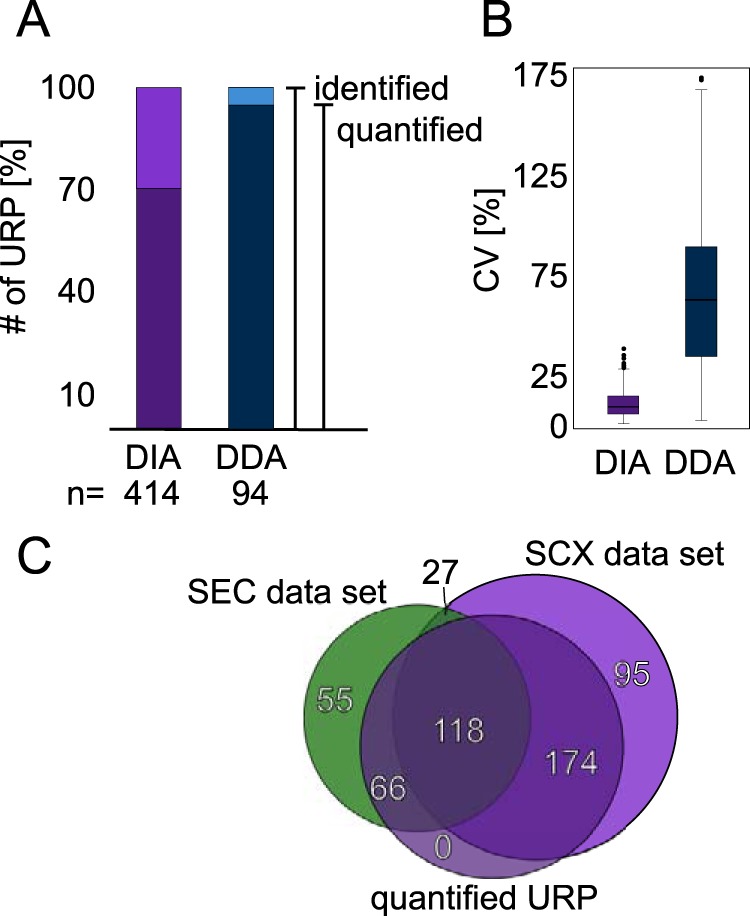
**Reproducibility of identification and quantitation of unique residue pairs (URPs).** *A*, Comparison of DIA (violet) versus *DDA* (blue) acquisition in terms of identified (light color) and quantified (dark color) unique residue pairs in triplicates. *B*, Comparison of DIA (light purple) and DDA (blue) in terms of CV values of peak areas. Box plot whiskers extend 1.5 times the interquartile range. *C*, Venn diagram of unique residue pairs from SCX data set compared with a foreign SEC data set and quantified residue pairs.

**Fig. 4.**
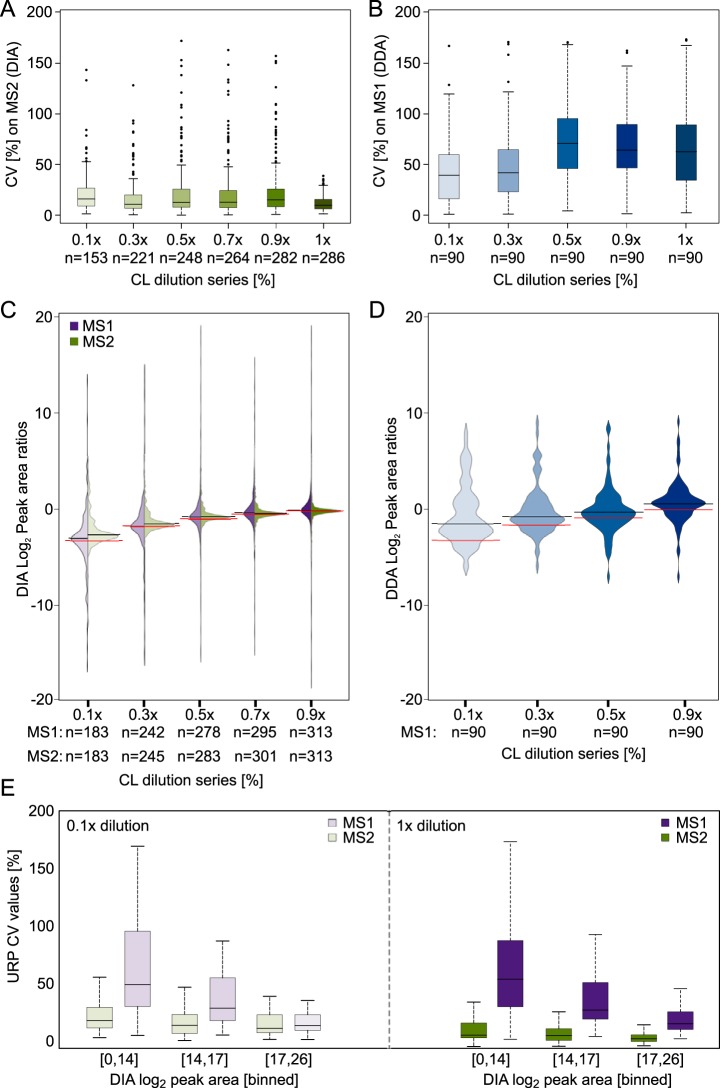
**Reproducibility of quantified unique residue pairs comparing data-independent and data-dependent acquisition.** *A*, Coefficient of variation (CV) within triplicates on MS2 level from median peak areas in % for each dilution step after quantitation (DIA), showing the reproducibility of label-free quantitation of peak areas. Box plot whiskers extend 1.5 times the interquartile range. *B*, Coefficient of variation (CV) within triplicates on MS1 level from the DDA analysis, median peak areas in % for each dilution step. Box plot whiskers extend 1.5 times the interquartile range *C*, Log_2_ peak area ratios of each dilution step, showing precision of mixing (black line) *versus* expected ratios (red line) on MS2 (green) and MS1 (purple) level. *D*, Log_2_ peak area ratios of each dilution step of DDA analysis, showing precision of mixing (black line) versus expected ratios (red line) on MS1 level (blue). *E*, CV from binned log_2_ peak areas in % of the 0.1x and 1x dilution (DIA), showing anticorrelation between residue pair peak areas and CV values (MS1: purple, MS2: green).

To increase the confidence in our quantitation results and to simplify the evaluation of the data set, for each dilution series we only included residue pairs that were quantifiable across the full set of respective triplicates. Using “match between runs” in Spectronaut enabled peptides to be quantified across replica even without being initially detected in every single replica. In the 1x sample, this yielded 292 (70%) quantified URPs out of 414 identified URPs. The same injection amount (1.5 μg) of HSA, albeit alone and not in mixture with six other proteins, analyzed by DDA led to 90 (95%) of 94 identified URPs being quantified across triplicate injections of a dilution series (0.1×, 0.3×, 0.5×, 0.9×, 1×). This means that DDA produced a higher quantified-to-identified ratio relative to DIA (Fig. 3*A*), at least when relying on the automated quantitation in Skyline. In how far this quantification is reliable will be investigated below. Interestingly, upon adding the foreign SEC data set (121 URPs) to the library, 66 unique residue pairs could be additionally quantified (Fig. 3*C*). The success rate of quantification was lower for this data (55%). Nevertheless, DIA allowed cross-links that had not been identified from a very extensive set of DDA acquisitions during library generation to be quantified. The overall success rate was 67% (358 quantified in at least one set of triplicates out of 535 URPs in the combined library). This relative proportions of URPs across the individual dilution samples were: 62% (1×), 62% (0.9×), 58% (0.7×), 54% (0.5×), 47% (0.3×), and 35% (0.1×). Peak area variation is represented using a coefficient of variation (CV) for each set of triplicates in the dilution experiment (Fig. 4*A*). The higher a CV value, the more variation was introduced during acquisition between peak areas of all replica pertaining to conditions. As one would expect, the lowest CV value is found for 1× dilution (CV: 10%). The CV value of the other dilutions ranges from 12 to 15% (0.1: 15%, 0.3: 12%, 0.5: 13%, 0.7: 12%, 0.9: 15%). This compares favorably with DDA, where only a single protein (HSA) was analyzed with a CV value of 66% for the 1× dilution (Fig. 3*B*). CVs of the other dilutions range from 42–76% (0.1: 42%, 0.3: 50%, 0.5: 76%, 0.9: 68%) (Fig. 4*B*). It compares also favorably to a previous DDA study using HSA, same cross-linker chemistry and manual curation of the whole data set, which resulted in a CV of 14% for the 1× (undiluted) sample (35). The reliability of DIA is further underpinned considering that a higher sample complexity increases the dynamic range problem and therefore impacts quantitation negatively.

Although CV values are in the same range for each DIA dilution experiment, a anticorrelation within each experiment between residue pair peak areas and CV values are clearly shown for undiluted as well as 0.1× dilution samples (Fig. 4*E*). This was also previously shown for DDA cross-linking data (35). This is consistent with the expectation that low intense features are less accurately quantified.

We then assessed how well ratios between samples could be determined in our dilution series. Based on MS2 peak areas, the dilution series could be clearly revealed. However, at high dilutions, the apparent dilution was smaller than the true dilution, for example, 0.1× appeared as only 0.137× (Fig. 4*C*). This ratio compression is presumably related to low MS2 peak intensities and the contribution of noise. Changing to MS1 level, which is characterized by higher signal intensities and higher S/N ratio, the apparent dilutions matched the actual dilutions in this data set more closely (0.1× looked like 0.104×). Contrary to DIA, the DDA dilution series matches poorly the expected ratios, even the 0.9x dilution shows clearly a ratio compression (Fig. 4*D*). This agrees with the high conditional CV values, which represent less reproducibility within each triplicate of each dilution experiment.

##### Label-free Quantitation of Cross-linked Peptides in a Complex Background

**Fig. 5.**
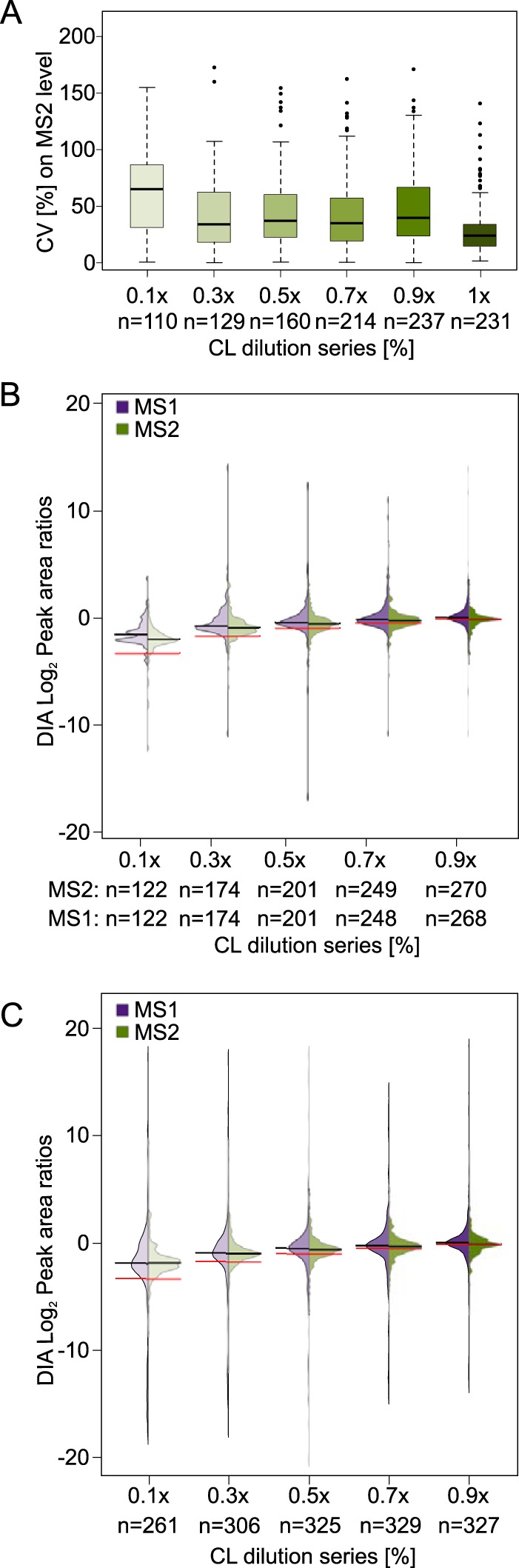
**Reproducibility of quantitation of unique residue pairs in a dilution series in a matrix of tryptic *E. coli* lysate and using shortened acquisition time.** *A*, Coefficient of variation (CV) on MS2 level from median peak areas in % for each dilution step after quantitation, showing the reproducibility of label-free quantitation of peak areas. Box plot whiskers extend 1.5 times the interquartile range. *B*, Log_2_ peak area ratios of each dilution step, showing precision of mixing (black line) *versus* expected ratios (red line) on MS2 (green) and MS1 (purple) level. *C*, Log_2_ peak area ratios of each dilution step, showing precision of mixing (black line) *versus* expected ratios (red line) on MS2 (green) and MS1 (purple) level using interference correction option in Spectronaut and a *E. coli* background library.

Cross-linked peptides are generally of low abundance because of substoichiometric cross-linking and often, multiple cross-link options for individual residues. This makes the detection of cross-linked peptides especially challenging as sample complexity increases. To probe the limits of our DIA-QCLMS workflow, we used tryptic *E. coli* lysate as a complex matrix, added to our dilution series at a 1:1 wt/wt ratio for the 1× dilution and respectively in 10-fold excess for our 0.1× dilution sample. An additional challenge to using the same DIA method as for the original dilution series was that the gradient time was shortened from 150 min to 85 min resulting in 7 (MS1) and 2 (MS2) data points per peak on average. As one would expect, this reduced the success rate at which URPs could be quantified across all samples (58%, down from 67% across 18 samples). CV values also deteriorated to 24% for 1:1 comparisons and 34–64% for the other dilution steps (Fig. 5*A*). To determine whether changes in abundance of cross-linked residue pairs were still distinguishable, MS1 and MS2 peak area ratios were plotted as log_2_ (Fig. 5*B*). MS1 and MS2 peak areas are very similar but differ notably in their expected ratios (red lines in violin plot), likely as a result of interferences from the increased sample complexity. This would also explain the higher CV values. Increasing complexity of samples therefore poses a challenge to quantitation success. Another explanation could be an interference effect caused by co-eluting precursors from the *E. coli* background (69). Adding a background library to our cross-link library and enabling interference correction in Spectronaut, shifted the MS2 peak area distribution of cross-linked peptides toward low intense peak areas (supplemental Fig. S1*i*), without differences in MS1 or CV values compared with disabling interference correction (supplemental Fig. S1h, S1*g*). Although this distribution shift might have led to a closer match of peak area ratios to the expected ratios, the effect of interference correction is not noticeable after summing up cross-link peptides to unique residue pairs (Fig. 5*C*). We wondered in how far summing up cross-linked peptides to residue pairs influences ratio distortion. Comparing data of the *E. coli* dilution series with and without the Top3 approach, often used in linear proteomics (70), showed an increase in ratio compression on MS1 (supplemental Fig. S2*a*) as well as MS2 level (supplemental Fig. S2*b*). In our case, using all available cross-linked peptides reduced ratio compression compared with using just the three most intense ones when quantifying residue pairs.

However, many cross-linked residue pairs could be quantified, and their abundances were distinguishable between different concentrations. Soon, the quantitation accuracy is likely to further improve and any dependence on manual data curation is likely to further decrease. For example, Spectronaut 12 has made progress on interference correction compared with the Spectronaut 11 version used here (see release notes). With enough awareness of the challenges connected to complex mixture analysis, it seems possible to detect differentially abundant cross-linked peptides in complex mixtures and hence to successfully conduct DIA-QCLMS under such conditions.

## CONCLUSION

Adapting Spectronaut for QCLMS analyses has extended the efforts of our laboratory to expand established proteomic quantitation software to cross-linking data, as done previously with MaxQuant (71) and Skyline (35), following initial proof-of-principle tests for QCLMS (24). Biognosys has added cross-linking data to the set of experiments that are automatically tested in each weekly build of Spectronaut to sustain optimal support for cross-linked data in Spectronaut in the future. The high accuracy that could be achieved, and especially the low CV at 1:1, suggest that even small changes in protein states could be picked up by QCLMS. DIA and Spectronaut significantly widen the scope of potential scientific applications of QCLMS and makes the analysis of structural states of large protein complexes or even cellular structures now appear possible.

## DATA AVAILABILITY

The mass spectrometry raw files, peak lists and the result files from xiFDR, xiDIA-library and Spectronaut used in this study have been deposited to the ProteomeXchange Consortium via the PRIDE partner repository with the dataset identifier PXD011036 (http://proteomecentral.proteomexchange.org/cgi/GetDataset?ID=PXD011036).

## Supplementary Material

Supplemental Material

Spectral library

Spectral library

Spectral library

Quantitation result file

Quantitation result file

Quantitation result file

XiFDR result file

XiFDR result file

XiFDR result file

## Footnotes

^1^ The abbreviations used are:

CLMS: cross-linking mass spectrometry
AcH: acetic acid
ACN: acetonitrile
AGC: automatic gain control
BS^3^: bis[sulfosuccinimidyl] suberate
CL: cross-linking
CV: coefficient of variation
DDA: data-dependent acquisition
DIA: data-independent acquisition
DTT: dithiothreitol
HCD: high energy collision dissociation
HAS: human serum albumin
IAA: 2-iodoacetamide
iTRAQ: isobaric tags for relative and absolute quantitation
LC-MS: liquid chromatography-mass spectrometry
LFQ: label-free quantitation
NH4Ac: ammonium acetate
PRM: parallel reaction monitoring
PSM: peptide spectrum matches
QCLMS: quantitative cross-linking mass spectrometry
SCX: strong cation exchange chromatography
SILAC: stable isotope-labelled amino acids
SRM/MRM: selected reaction monitoring/multiple reaction monitoring
TMT: tandem mass tag
URPs: unique residue pairs.
