# Supplementary material for: Data-independent Acquisition Improves Quantitative Cross-linking Mass Spectrometry
Source: Mol Cell Proteomics. 2019 Jan 16;18(4):786–95. doi: 10.1074/mcp.TIR118.001276 (PMC6442367; doi:10.1074/mcp.TIR118.001276)
Supplement: Supplemental Material [file 142541_0_supp_249977_pjmkyw.pdf]

Data-independent acquisition improves quantitative cross-linking mass spectrometry

Fränze Müller[1], Lars Kolbowski[1,2], Oliver M. Bernhardt[3], Lukas Reiter[3], Juri Rappsilber[1,2,*]

[1] Bioanalytics, Institute of Biotechnology, Technische Universität Berlin, 13355 Berlin, Germany
[2] Wellcome Centre for Cell Biology, School of Biological Sciences, University of Edinburgh, Edinburgh EH9 3BF, Scotland, United Kingdom
[3] Biognosys, 8952 Zürich-Schlieren, Switzerland

Supplemental Material:

Supplemental Figure S1 – Performance comparison of Spectronaut 11 and 12 for automated and manual curation of the 7 protein mix dilution series with and without *E. coli* as background matrix.

Supplemental Figure S2 – Comparison of Top3 approach vs. summing up all cross-linked peptides for residue pair level in a quantitative cross-linking mass spectrometry analysis.

XiFDR_PSMfile_7PMix_BS3_5%linklevel.xls - List of all peptide sequences identified, Precursor charge, m/z, all modifications observed, sites of modification within each peptide, peptide identification scores, linkage side and linkage position after FDR estimation (5%link level) for the pure dilution series of the 7 protein mix cross-linked with BS^3.

XiFDR_PSMfile_7PMix_BS3_ecolibg_5%linklevel.xls - List of all peptide sequences identified, Precursor charge, m/z, all modifications observed, sites of modification within each peptide, peptide identification scores, linkage side and linkage position after FDR estimation (5%link level) for the dilution series of the 7 protein mix cross-linked with BS^3 using *E.coli* as background matrix.

XiFDR_PSMfile_HSA_BS3_5%linklevel.xls - List of all peptide sequences identified, Precursor charge, m/z, all modifications observed, sites of modification within each peptide, peptide identification scores, linkage side and linkage position after FDR estimation (5%link level) for the dilution series of human serum albumin (HSA) cross-linked with BS^3.

XiDIALib_7PMix_BS3_ecolibg_nCL10_nLin10_lossyFalse.xls – Spectral library for the 7 protein mix dilution series using *E. coli* as background matrix.

XiDIALib_7PMix_BS3_nCL10_nLin10_lossyFalse.xls - Spectral library for the pure dilution series of the 7 protein mix

HSA_BS3_DDA_dilution.xls - Spectral library for the HSA DDA dilution series.

E.coli_background_library.xls – Background spectral library of *E. coli* lysate.

Spectronaut_output_7PMix_BS3_SCX_ingel.xls – Quantitation report file from Spectronaut for the pure dilution series of the 7 protein mix

Spectronaut_output_7PMix_BS3_SCX_ingel_ecolibg.xls - Quantitation report file from Spectronaut for the dilution series of the 7 protein mix using *E. coli* as background matrix.

7PMix_BS3_SCX_E.coli_bg_lib_withI_Report.xls - Quantitation report file from

Spectronaut for the dilution series of the 7 protein mix using *E. coli* as background matrix and additional *E. coli* background library with interference correction option.

HSA_BS3_DDA_dilution_series_Skyline.xls - Quantitation report file from Skyline for the dilution series of the HSA cross-linked with BS^3 (DDA data).

Figure S1

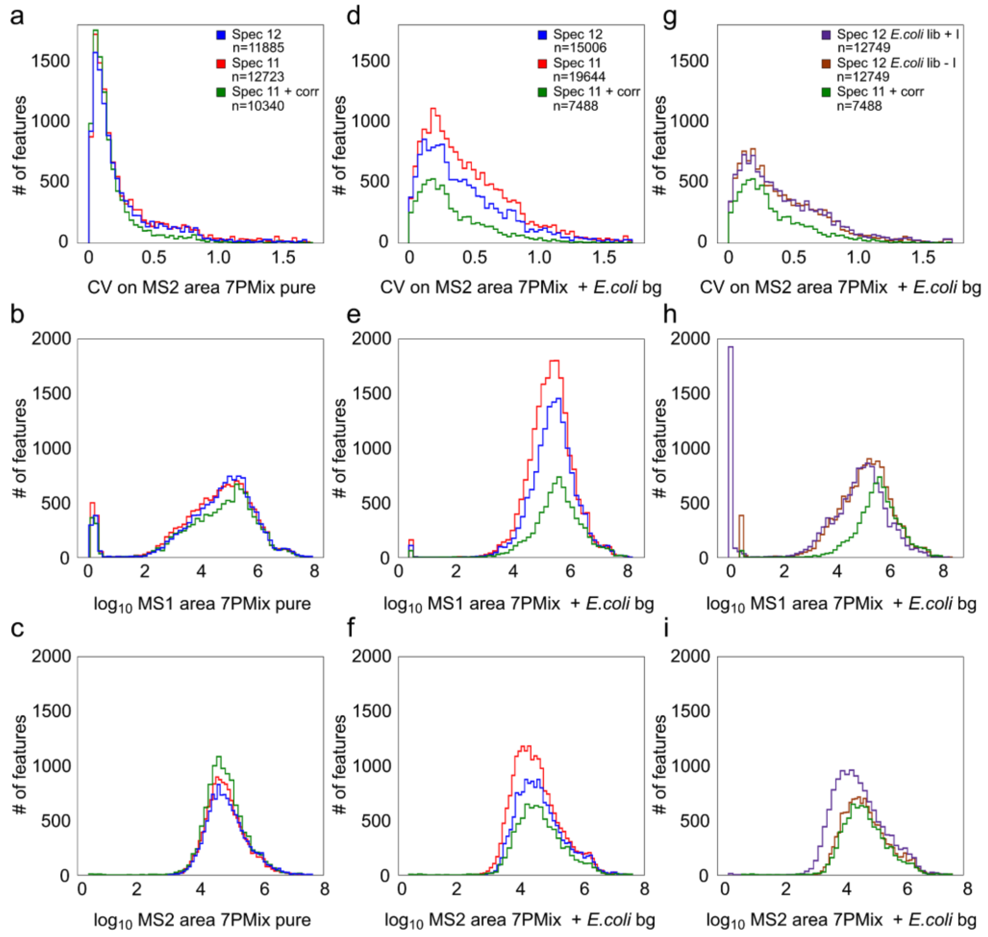

Fig. S1: *Performance comparison between Spectronaut 11 and 12 for a cross-linking/mass spectrometry analysis. a: histogram of CV values for the pure dilution series (blue: automated QCLMS analysis in Spectronaut 12, red: automated QCLMS analysis in Spectronaut 11, green: manual corrected dataset in Spectronaut 11). b: histogram of log_{10} MS1 peak areas for cross-linked features in the pure dilution series (blue: automated QCLMS analysis in Spectronaut 12, red: automated QCLMS analysis in Spectronaut 11, green: manual corrected dataset in Spectronaut 11). c: histogram of log_{10} MS2 peak areas for cross-linked features in the pure dilution series (blue: automated QCLMS analysis in Spectronaut 12, red: automated QCLMS analysis in Spectronaut 11, green: manual corrected dataset in Spectronaut 11). d: histogram of CV values for the dilution series using E. coli as background (blue: automated QCLMS analysis in Spectronaut 12, red: automated QCLMS analysis in Spectronaut 11, green: manual corrected dataset in Spectronaut 11). e: histogram of log_{10} MS1 peak areas for cross-linked features in the dilution series using E. coli as background (blue: automated QCLMS analysis in Spectronaut 12, red: automated QCLMS analysis in Spectronaut 11, green: manual corrected dataset in Spectronaut 11). f: histogram of log_{10} MS2 peak areas for cross-linked features in the dilution series using E. coli as background (blue: automated QCLMS analysis in Spectronaut 12, red: automated QCLMS analysis in Spectronaut 11, green: manual corrected dataset in Spectronaut 11).g: histogram of CV values for the dilution series using E. coli as background (violet: automated QCLMS analysis in Spectronaut 12 using interference correction and an E. coli background library, brown: automated QCLMS analysis in Spectronaut 12 without interference correction using an E. coli background library, green: manual corrected dataset in Spectronaut 11). h: histogram of log_{10} MS1 peak areas for cross-linked features in the dilution series using E. coli as background (violet: automated QCLMS analysis in Spectronaut 12 using interference correction and an E. coli background library, brown: automated QCLMS analysis in Spectronaut 12 without interference correction using an E. coli background library, green: manual corrected dataset in Spectronaut 11). i: histogram of log_{10} MS2 peak areas for cross-linked features in the dilution series using E. coli as background (violet: automated QCLMS analysis in Spectronaut 12 using interference correction and an E. coli background library, brown: automated QCLMS analysis in Spectronaut 12 without interference correction using an E. coli background library, green: manual corrected dataset in Spectronaut 11). Note that the number of features decreases form Spectronaut 11 to Spectronaut 12 due to improved internal FDR estimation. Less features are passing the q-value filtering in Spectronaut 12. The number of features quantified decreases for the corrected dataset due to manual curation and excluding low quality features from the analysis.*

Figure S2

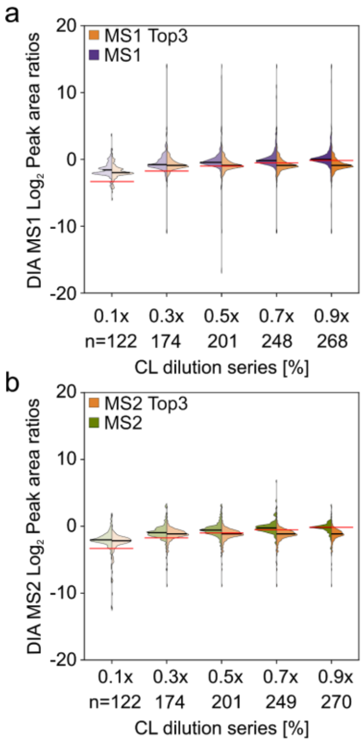

Fig. S2: *Comparison of Top3 approach vs. summing up all cross-linked peptides for residue pair level in a quantitative cross-linking mass spectrometry analysis. a: Log_2 peak area ratios of each dilution step, showing precision of mixing (black line) vs expected ratios (red line) on MS1 without Top3 approach (purple) and using Top3 (orange). b: Log_2 peak area ratios of each dilution step, showing precision of mixing (black line) vs expected ratios (red line) on MS2 without Top3 approach (green) and using Top3 (orange).*
